# 基于SEER数据库构建原发性纵隔生殖细胞瘤患者生存预测的列线图

**DOI:** 10.3779/j.issn.1009-3419.2023.102.11

**Published:** 2023-03-20

**Authors:** Zuqiang ZHENG, Zhongjie WU, Yi HU, Yanfei ZHANG, Congyi DING, Xinkai ZOU

**Affiliations:** ^1^310053 杭州，浙江中医药大学（郑祖强，丁聪毅，邹昕锴）; ^1^Zhejiang Chinese Medical University, Hangzhou 310053, China; ^2^314001 嘉兴，嘉兴市第一医院心胸外科（郑祖强，吴中杰，胡奕，张雁飞，丁聪毅，邹昕锴）; ^2^Department of Cardiothoracic Surgery, Jiaxing First Hospital, Jiaxing 314001, China

**Keywords:** 原发性纵隔生殖细胞瘤, 预后因素, SEER数据库, 疾病特异性生存期, 列线图, Primary mediastinal germ cell tumor, Prognostic factors, Surveillance, Epidemiology, and End Results database, Disease special survival, Nomogram Lasso regression

## Abstract

**背景与目的** 原发性纵隔生殖细胞瘤（primary mediastinal germ cell tumor, PMGCT）是一种相对罕见且偶尔会具有高度侵袭性的纵隔肿瘤。目前对PMGCT的疾病特异性生存期（disease special survival, DSS）的相关研究报道较少，大数据分析亦相对较少，DSS预后模型也较为少见。本研究旨在探讨影响PMGCT DSS的预后相关因素，并构建简便、有效、可对PMGCT患者DSS预后情况进行预测的列线图。**方法** 回顾性分析从监测、流行病学和最终结果（Surveillance, Epidemiology, and End Results, SEER）数据库提取的1975年-2019年共347例PMGCT患者的临床病理资料。采用Kaplan-Meier法及Log-rank检验估计DSS。执行Cox比例风险回归模型筛选影响预后的独立危险因素，构建个体化列线图预测PMGCT患者的3年、5年、8年DSS。通过受试者工作特征（receiver operating characteristic, ROC）曲线、校正曲线及决策曲线分析（decision curve analysis, DCA）评估模型的预测精度。**结果** PMGCT患者的3年、5年、8年生存率分别为84.6%、83.6%、83.3%。单因素Cox回归分析显示组织学分型、手术与否、年龄、肿瘤大小、肿瘤转移情况及肿瘤分期6项因素可影响PMGCT的预后（P<0.05），多因素Cox回归分析显示组织学分型、手术与否、年龄、肿瘤大小是PMGCT患者预后的独立危险因素（P<0.05），利用这些独立危险因素构建了列线图模型。ROC的曲线下面积（area under the curve, AUC）为0.824，3年、5年、8年生存时间的校正曲线以及DCA曲线，三者结果提示本研究的列线图评估预测结果与真实结果之间有良好的一致性。**结论** PMGCT中组织学分型为精原细胞瘤的患者比非精原细胞瘤患者预后更佳，年龄>40岁、肿瘤大小≥15 cm且未进行过手术治疗的患者预后不佳。列线图模型可以对PMGCT患者的DSS进行准确直观的预测。

## Abstract (en)

**Background and objective** Primary mediastinal germ cell tumor (PMGCT) is a rare but occasionally highly invasive mediastinal tumor. At present, there are few related disease special survival (DSS) studies on PMGCT, rare large data analysis, and uncommon DSS prognostic models. This study was to investigate the prognostic factors of DSS of the PMGCT patients, and build a simple and effective nomogram to predict the DSS prognosis in patients with PMGCT. **Methods** Retrospective clinicopathological data of 325 patients with PMGCT from 1975 to 2019 were extracted from Surveillance, Epidemiology, and End Results (SEER) database. The Kaplan-Meier method along with the Log-rank test were utilized to estimate the DSS. Cox proportional hazard regression model was used to screen the independent risk factors affecting prognosis, from which an individualized nomogram was constructed to predict 3-yr, 5-yr and 8-yr DSS of patients with PMGCT. The prediction accuracy of the model is evaluated by receiver operating characteristic (ROC) curve, correction curve and decision curve analysis (DCA) curve. **Results** The 3-yr, 5-yr and 8-yr survival rates of PMGCT were 84.6%, 83.6% and 83.3%, respectively. Univariate analysis showed that histology, surgery, age, tumor size, metastasis and stage could affect the prognosis of PMGCT. Multivariate analysis showed that histology, surgery, age and tumor size were independent risk factors for the prognosis of PMGCT patients, and the nomogram was constructed using these independent risk factors. The area under the curve (AUC) of ROC curve was 0.824. The results of the correction curves of 3-yr, 5-yr and 8-yr survival time and DCA, indicated that there was a good consistency between the predicted results of the nomogram evaluation and the real results. **Conclusion** Patients with histological classification of seminoma in PMGCT have a better prognosis than patients with non-seminoma. The prognosis of patients with over the age of 40 yr, tumor size ≥15 cm and without surgical treatment was even worse. The nomogram model can accurately and intuitively predict the DSS of patients with PMGCT.

原发性纵隔生殖细胞瘤（primary mediastinal germ cell tumor, PMGCT）是一种最常见的性腺外生殖细胞瘤，占生殖细胞瘤的1%-3%、纵隔肿瘤的4%、性腺外生殖细胞瘤的48.1%^[1⇓-3]^。PMGCT相对较为罕见，关于其临床特征及疾病特异性生存期（disease special survival, DSS）影响因素的研究较少。监测、流行病学和最终结果（Surveillance, Epidemiology, and End Results, SEER）数据库拥有大量的临床回顾性资料，便于评估影响PMGCT预后的危险因素。列线图是对疾病预后危险因素进行综合分析，并进行可视化、个体化评估的方法。本研究运用SEER数据库数据，旨在探讨影响PMGCT的DSS预后相关因素，并构建简便、有效的预测列线图，为临床医生评估PMGCT预后情况提供参考依据。

## 1 资料与方法

### 1.1 数据来源

通过SEER*Stat version 8.3.5软件（v8.3.5, https://seer.cancer.gov/seerstat/）提取SEER数据库（http://seer.cancer.gov/）中PMGCT病例数据。将PMGCT数据作为研究对象，DSS或末次随访时间作为研究结局，提取条件是诊断年份（1975年-2019年）、肿瘤原发位置（纵隔）、组织学类型（生殖细胞瘤），提取变量包括患者ID、生存时间、生存状态、死因分类、癌症与非癌症死亡原因、确诊方法、手术信息、年龄、肿瘤直径、远处转移、分期、既往肿瘤病史、人种、性别、放疗信息、化疗信息、婚姻状况。纳入标准：（1）病理学诊断为生殖细胞瘤；（2）肿瘤原发位置为纵隔；（3）有完整的随访信息；（4）确诊方法：病理诊断；（5）死亡原因：PMGCT。排除标准：（1）30天内死亡者；（2）各变量有缺失者；（3）随访信息不完整者。SEER数据库中查找符合提取条件的病例，共获得1,462例患者信息。检索生存时间≥1个月者；生存状态信息完整者；死因分类中为存活或死于癌症者；癌症与非癌症死亡原因变量中为存活或死于纵隔疾病者；确诊方法为病理确诊者，将上述五者取交集共获得910例PMGCT患者DSS资料。最后排除研究变量中信息缺失者，共获得完整有效信息数据347例。

对获取的PMGCT患者的SEER数据库数据进行整理分组：（1）依据ICD-O-3分类标准，将数据库中PMGCT组织学类型由代码翻译为可分析分型。将9060/3（无性细胞瘤）、9065/3（非精原细胞瘤样生殖细胞瘤）、9070/3（胚胎性癌）、9071/3（卵黄囊瘤）、9080/3（恶性畸胎瘤）、9085/3（混合生殖细胞瘤）、9100/3（绒毛膜癌）、9101/3（绒毛膜癌与其他生殖细胞成分结合）归纳为非精原细胞瘤（non-seminoma）；将9061/3（精原细胞瘤）归纳为精原细胞瘤（seminoma）；将9064/3（生殖细胞瘤）归纳为未分类生殖细胞瘤（germ cell tumor-NOS）；原发于纵隔的精原细胞瘤和非精原细胞瘤分别称为原发性纵隔精原细胞瘤（primary mediastinal seminoma, PMSGCT）和原发性纵隔非精原细胞瘤（primary mediastinal non-seminoma, PMNSGCT）；（2）将手术情况分为未进行手术和手术两组；（3）将年龄分为≤21岁、22岁-40岁、>40岁三组；（4）由于肿瘤直径变量有40例登记为未知，根据文献，该变量对预后具有一定程度影响，故将肿瘤大小分为<15 cm、≥15 cm、未知三组；（5）将“骨转移、肝转移、肺转移、脑转移、淋巴结转移、其他转移”整合成肿瘤转移情况（metastasis），并将其分为转移和未转移两组；（6）在SEER数据库中，将肿瘤分期（combined summary stage）简单地划分为局部（局限于起源的腺体或局限性的侵袭性癌）、区域性（邻近结缔组织）和远处（邻近的器官/结构或进一步的传染性扩展或任何阳性淋巴结）以及登记为未知四种，因此我们将肿瘤分期分为远处扩展（Distant）、未发生远处扩展（Non-distant）、未知（Unknown）三组；（7）将既往肿瘤病史分为有、无两组；（8）将区域淋巴结清扫术（regional lymph node surgery）分为有、无两组；（9）人种变量中共有白人（white, W）、黑人（black, B）、亚洲或太平洋岛民（Asian or Pacific Islander, A）、美洲印地安人或阿拉斯加原住民（American Indian/Alaska Native, AI）四个亚分类，由于A与AI病例数较少，故将人种分为B、W、其他三组；（10）将有效数据的肿瘤原发位置代码转换成可分析分型。C37.9-胸腺、C38.1-前纵隔、C38.2-后纵隔、C38.3-纵隔未分类分别作为胸腺（thymus）、前纵隔（anterior mediastinum）、后纵隔（posterior mediastinum）、纵隔位置未分类（mediastinum-NOS）四组；（11）将性别分为女性和男性两组；（12）由于SEER数据库自身局限性，对放化疗信息采集模糊，故无法明确区分未做放化疗还是放化疗信息未知。因此保留数据库中原有表述，将放疗信息分为No/Unknown和Yes两组，将化疗信息分为No/Unknown和Yes两组；（13）将婚姻状况分为单身、结婚、未知三组。

### 1.2 统计分析

采用R软件（4.1.3版本）。对组织学分型、手术与否、年龄、肿瘤大小、肿瘤转移情况、分期、既往肿瘤病史、区域淋巴结手术、人种、肿瘤原发位置、性别、放疗、化疗、婚姻状况采用Kaplan-Meier法计算研究对象的DSS，并用Log-rank检验比较DSS差异。使用Cox比例风险回归模型评估临床病理信息和生存时间之间的关系。通过单因素Cox回归分析，筛选出具有统计学意义的临床病理信息。将具有统计学意义的单因素Cox回归分析变量纳入多因素Cox回归分析，得到独立的预后影响因子，并在此基础上构建一个列线图DSS生存的预后模型。采用受试者工作特征（receiver operating characteristic, ROC）曲线、校正曲线和决策曲线分析（decision curve analysis, DCA）对列线图生存预后模型的准确性进行评价^[4]^。P<0.05为差异具有统计学意义。

## 2 结果

### 2.1 PMGCT患者人口统计学和基线特征

PMGCT患者的3年、5年、8年DSS分别为84.6%（95%CI: 82.2%-87.0%）、83.6%（95%CI: 81.1%-86.1%）、83.3%（95%CI: 80.8%-85.8%）。值得注意的是，在SEER数据库中，由于其自身局限性，在采集患者放化疗信息方面，无法准确判断患者未做放化疗还是该信息未知。肿瘤原发位置的胸腺及后纵隔分类、性别中的女性分类、化疗中的No/Unknown分类、婚姻状况中的未知分类，纳入的数据均未发生结局事件（表1）。

**表1:** PMGCT患者的人口统计学和基线特征

Variables	Survival (n=318)	Death (n=29)	Overall (n=347)
Histology			
Non-seminoma	168 (52.8%)	24 (82.8%)	192 (55.3%)
NOS	46 (14.5%)	4 (13.8%)	50 (14.4%)
Seminoma	104 (32.7%)	1 (3.4%)	105 (30.3%)
Surgery			
No	142 (44.7%)	19 (65.5%)	161 (46.4%)
Yes	176 (55.3%)	10 (34.5%)	186 (53.6%)
Age (yr)			
≤21	79 (24.8%)	7 (24.1%)	86 (24.8%)
22-40	169 (53.1%)	10 (34.5%)	179 (51.6%)
>40	70 (22.0%)	12 (41.4%)	82 (23.6%)
Tumor size (cm)			
<15	206 (64.8%)	13 (44.8%)	219 (63.1%)
≥15	72 (22.6%)	11 (37.9%)	83 (23.9%)
Unknown	40 (12.6%)	5 (17.2%)	45 (13.0%)
Metastasis			
No	280 (88.1%)	19 (65.5%)	299 (86.2%)
Yes	38 (11.9%)	10 (34.5%)	48 (13.8%)
Stage			
Distant	57 (17.9%)	11 (37.9%)	68 (19.6%)
Non-distant	251 (78.9%)	17 (58.6%)	268 (77.2%)
Unknown	10 (3.1%)	1 (3.4%)	11 (3.2%)
Previous history of malignancy			
No	294 (92.5%)	25 (86.2%)	319 (91.9%)
Yes	24 (7.5%)	4 (13.8%)	28 (8.1%)
Regional lymph node surgery			
No	253 (79.6%)	26 (89.7%)	279 (80.4%)
Yes	65 (20.4%)	3 (10.3%)	68 (19.6%)
Race			
Black	25 (7.9%)	1 (3.4%)	26 (7.5%)
Other	53 (16.7%)	1 (3.4%)	54 (15.6%)
White	240 (75.5%)	27 (93.1%)	267 (76.9%)
Primary tumor site			
Thymus	12 (3.8%)	0 (0.0%)	12 (3.5%)
Anterior mediastinum	182 (57.2%)	12 (41.4%)	194 (55.9%)
Posterior mediastinum	2 (0.6%)	0 (0.0%)	2 (0.6%)
Mediastinum-NOS	122 (38.4%)	17 (58.6%)	139 (40.1%)
Gender			
Female	18 (5.7%)	0 (0.0%)	18 (5.2%)
Male	300 (94.3%)	29 (100.0%)	329 (94.8%)
Radiation			
No/unknown	301 (94.7%)	26 (89.7%)	327 (94.2%)
Yes	17 (5.3%)	3 (10.3%)	20 (5.8%)
Chemotherapy			
No/unknown	33 (10.4%)	0 (0.0%)	33 (9.5%)
Yes	285 (89.6%)	29 (100.0%)	314 (90.5%)
Marital status			
Married	112 (35.2%)	10 (34.5%)	122 (35.2%)
Single	190 (59.7%)	19 (65.5%)	209 (60.2%)
Unknown	16 (5.0%)	0 (0.0%)	16 (4.6%)

PMGCT: primary mediastinal germ cell tumor; NOS: not otherwise specified.

### 2.2 PMGCT患者DSS的Kaplan-Meier生存曲线

PMGCT患者DSS影响因素共有5个，分别是组织学分型、手术与否、年龄、肿瘤转移情况及肿瘤分期（P<0.05），而肿瘤大小（P=0.074）、既往肿瘤病史（P=0.140）、区域淋巴结手术（P=0.200）、人种（P=0.090）、肿瘤原发位置（P=0.110）、性别（P=0.180）、放疗（P=0.220）、化疗（P=0.066）、婚姻状态（P=0.460）未见显著差异。见图1、图2。

**图1:**
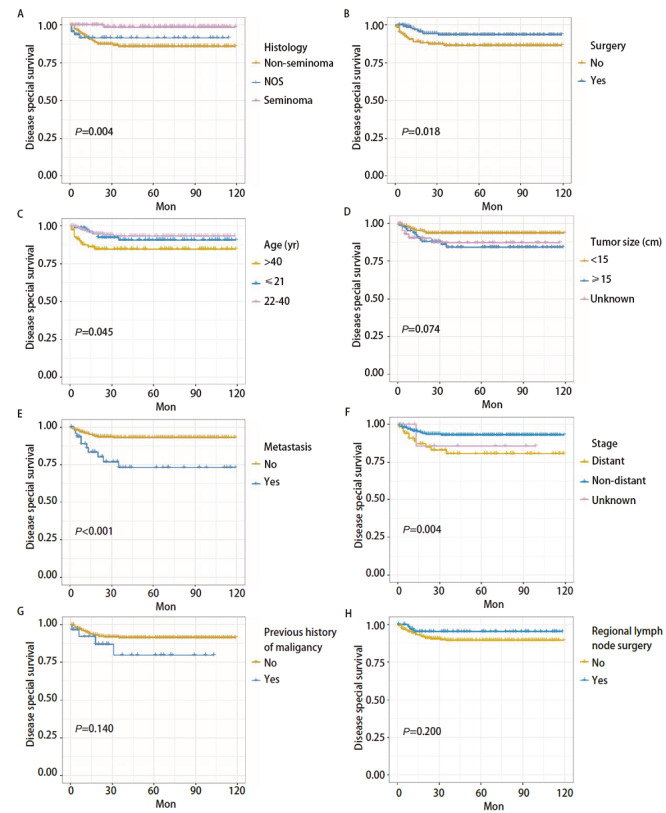
采用Kaplan-Meier法，根据组织学（A）、手术（B）、年龄（C）、肿瘤大小（D）、转移（E）、分期（F）、既往恶性肿瘤史（G）、区域淋巴结手术（H）预测PMGCT患者的DSS

**图2:**
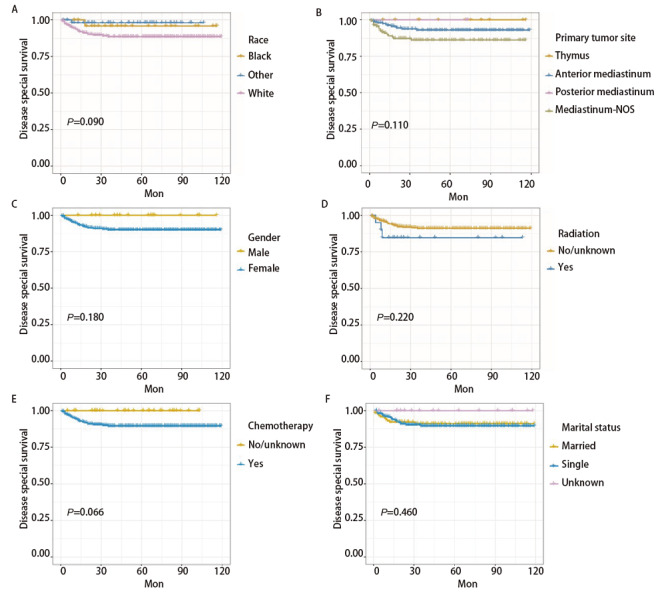
采用Kaplan-Meier法，根据种族（A）、原发肿瘤部位（B）、性别（C）、放疗（D）、化疗（E）、婚姻状况（F）预测PMGCT患者的DSS

### 2.3 单因素与多因素Cox回归分析

单因素Cox回归分析显示，组织学分型、手术与否、年龄、肿瘤大小、肿瘤转移情况及肿瘤分期（P<0.05）是PMGCT患者DSS的预后危险因素（表2）。其中肿瘤转移情况中发生转移的HR值为3.857（P<0.001），表明发生肿瘤转移比未发生肿瘤转移的患者死亡风险比率更高。多因素Cox回归分析（表3）显示组织学分型、手术与否、年龄、肿瘤大小是PMGCT患者预后的独立危险因素（P<0.05）。 进行过手术治疗的患者HR值为0.22（P<0.001），表明进行过手术治疗的患者远比未进行手术治疗的患者死亡风险率低，行手术治疗是保护性因素。放化疗、肿瘤原发位置、性别、婚姻状况的Kaplan-Meier生存曲线未见显著差异，因此未纳入Cox回归分析。

**表2:** 单因素Cox回归分析

Variables	HR	95%CI	P
Histology			
Non-seminoma	Reference		
NOS	0.726	0.252-2.093	0.553
Seminoma	0.074	0.010-0.544	0.011
Surgery			
No	Reference		
Yes	0.408	0.190-0.878	0.022
Age (yr)			
>40	Reference		
≤21	0.493	0.194-1.253	0.137
22-40	0.367	0.158-0.849	0.019
Tumor size (cm)			
<15	Reference		
≥15	2.352	1.054-5.251	0.037
Unknown	2.133	0.760-5.984	0.150
Metastasis			
No	Reference		
Yes	3.857	1.791-8.307	0.001
Stage			
Distant	Reference		
Non-distant	0.352	0.165-0.752	0.007
Unknown	0.616	0.079-4.768	0.642
Previous history of malignancy			
No	Reference		
Yes	2.171	0.755-6.242	0.150
Regional lymph node surgery			
No	Reference		
Yes	0.464	0.141-1.534	0.208
Race			
Black	Reference		
Other	0.523	0.033-8.368	0.647
White	2.967	0.403-21.835	0.286

**表3:** 多因素Cox回归分析

Variables	HR	95%CI	P
Histology			
Non-seminoma	Reference		
NOS	0.536	0.176-1.635	0.273
Seminoma	0.039	0.005-0.302	0.002
Surgery			
No	Reference		
Yes	0.220	0.091-0.527	0.001
Age (yr)			
>40	Reference		
≤21	0.598	0.223-1.605	0.307
22-40	0.394	0.167-0.931	0.034
Tumor size (cm)			
<15	Reference		
≥15	2.344	1.027-5.349	0.043
Unknown	1.374	0.462-4.082	0.568
Metastasis			
No	Reference		
Yes	2.785	0.329-23.551	0.347
Stage			
Distant	Reference		
Non-distant	1.805	0.222-14.654	0.580
Unknown	2.538	0.143-44.995	0.526

### 2.4 PMGCT生存时间预测模型的列线图

PMGCT列线图模型包含4个独立危险因素（组织学分型、手术与否、年龄、肿瘤大小）（图3）。在PMGCT列线图预后模型中，非精原细胞瘤组织学类型的赋值为100分，是占比最高的预后影响因素；未进行手术治疗的赋值约为48分；年龄>40岁者和肿瘤大小≥15 cm者分别赋值约为27.5分和26.25分，是占比较低的预后影响因素。假设PMGCT患者的组织学类型是非精原细胞瘤类型、未进行手术治疗、年龄65岁、肿瘤大小为15 cm，那么该患者的DSS预后赋值是100+48+27.5+26.25=201.75分。在列线图中，201.75分对应3年、5年、8年DSS的生存率在30%左右。

**图3:**
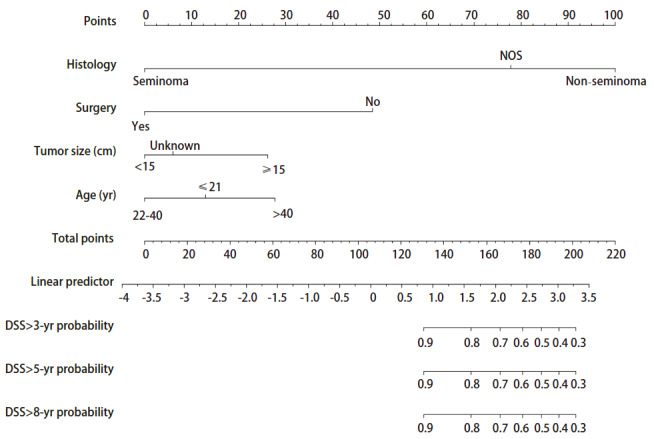
预测PMGCT患者DSS的预后列线图

### 2.5 PMGCT患者DSS预测模型列线图的校验

构建预测模型ROC曲线的曲线下面积（area under the curve, AUC）是0.824；构建模型的DCA曲线在右上象限，说明该模型具有较为精确的预测能力；3年、5年、8年生存时间的校正曲线靠近理性的45°虚线，提示该模型预测能力较好（图4）。

**图4:**
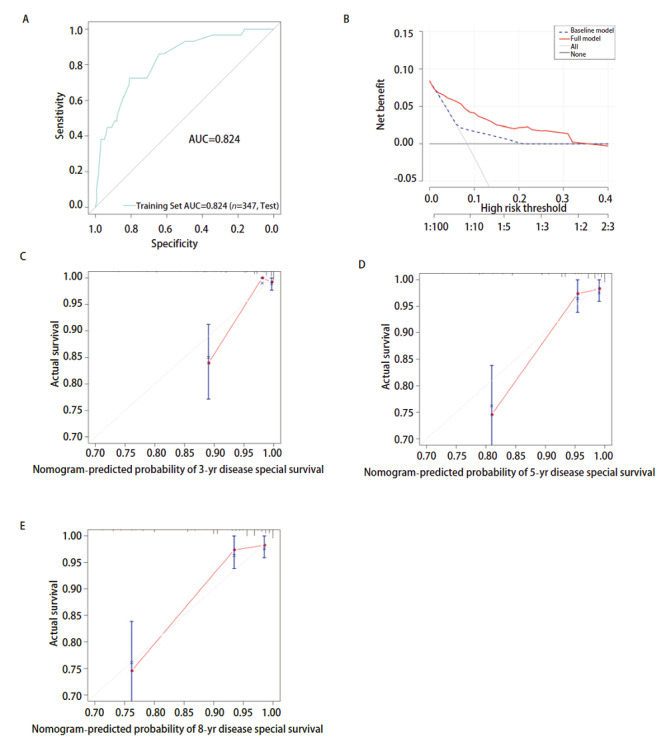
用于预测PMGCT患者DSS的ROC曲线、DCA曲线和校准曲线。 A：ROC曲线；B：DCA曲线；C：3年校正曲线；D：5年校正曲线；E：8年校正曲线。

## 3 讨论

本研究提取了SEER数据库中的347例PMGCT患者的临床资料，多因素Cox回归分析提示存在4个影响预后的独立危险因素，将其作为列线图的变量。通过ROC、校正曲线和DCA检验，本列线图显示出较好的准确度。生殖细胞瘤好发部位是睾丸，仅有约5%肿瘤位于性腺之外的部位，而性腺外生殖细胞瘤最常见的原发位置是纵隔^[5⇓-7]^。PMGCT目前有来自国际生殖细胞肿瘤合作组织（International Germ Cell Cancer Collaborative Group, IGCCCG）的基于预后的转移性生殖细胞瘤分期系统以及基于胸腺恶性肿瘤的Masaoka Koga分期系统，但均为非主流分期系统^[8⇓-10]^。性腺外生殖细胞瘤与生殖细胞瘤有相似之处，但在胚胎和遗传学上有它的特殊性。大多数学者认为，在胚胎发育过程中，睾丸的形成依赖于原始生殖细胞从近端外胚层迁移到背侧的尿生殖脊的过程^[11]^，部分生殖细胞在迁移的过程中发生了停滞，从而使纵隔具备了发生PMGCT的细胞基础。因为PMGCT组织学类型和生物学行为的不同，所以对PMGCT单独分析和预后模型的构建尤为重要。本列线图和以往的评估方法相比，其优势在于个体化和直观化，为临床医生提供了对个体PMGCT患者进行简便、有效预后判断的方法。

多因素Cox回归分析显示组织学分型、手术与否、年龄、肿瘤大小是影响PMGCT患者DSS的独立危险因素（P<0.05）。多项研究^[10,12⇓⇓-15]^表示，手术情况、组织学分型、肿瘤转移情况等对预后具有不同程度的影响。2021年，IGCCCG更新了转移性生殖细胞瘤的分类，开发了可以判断预后情况的模型，该模型提示年龄的增加、肺转移的存在和病理类型为非精原细胞瘤是PMGCT患者总生存期（overall survival, OS）的独立危险因素，是PMGCT的预后不良因素^[10]^。

多篇文献^[10,13,16,17]^报道，PMSGCT的预后好于PMNSGCT，其5年生存率保持在90%以上，而PMNSGCT的5年生存率维持在50%左右^[17⇓⇓⇓-21]^。本研究通过多因素回归分析发现，组织学分型对PMGCT的DSS具有极其重要的影响，精原细胞瘤的HR值为0.039（P=0.002），表明精原细胞瘤是PMGCT患者DSS的保护因素，患者往往拥有更好的生存期。

近年来，PMGCT以综合治疗为主^[22]^，根据病理类型采用不同的治疗方案。良性PMGCT主要是手术切除；恶性PMGCT则采用新辅助放化疗，并辅以手术治疗，因此手术在PMGCT的治疗中起到了重要作用。国内外研究^[12,14,15,23]^表明无论肿瘤选择姑息手术还是根治手术，均能不同程度有利于患者的预后。本项研究发现未进行手术治疗是PMGCT患者DSS的独立危险因素（P=0.001），在列线图中占有较大的分值。

在既往的国内外研究中，年龄和性别因素是否对预后有影响仍然存在争议^[8,10,14,18,24]^。在多因素Cox回归分析中，年龄>40岁相比22岁-40岁之间的PMGCT患者，预后不佳（P=0.019），该结果符合IGCCCG研究报道^[10]^。Kaplan-Meier分析结果表明性别（P=0.180）对PMGCT的DSS影响没有统计学意义。

Yang等^[15]^研究表明，当肿瘤大小>15 cm时，PMNSGCT患者预后不佳。本研究中，通过多因素Cox回归分析发现，肿瘤大小≥15 cm是PMGCT的预后不良因素（P=0.037），与上述学者研究结果类似。肿瘤大小≥15 cm的PMGCT患者预后不良，分析其原因可能有：纵隔具有一定空间，只有肿瘤长到一定程度后才会引起胸闷、胸痛、呼吸困难等临床症状，贻误治疗；肿瘤恶变导致肿块增大、预后不良等。具体原理和机制还需要进一步研究。

本研究根据上述预后独立危险因素，构建了预测较为准确的PMGCT患者DSS列线图。ROC曲线的AUC值是0.824，DCA曲线位于右上象限，3年、5年、8年DSS的校正曲线与理想的45°虚线相近，三者的结果均显示列线图预测的生存率和实际观测到的生存率一致性较好。

与以往大多数PMGCT回顾性分析和构建预后模型研究相比，本研究的优势在于通过SEER数据库较为庞大的临床资料，首次研究和分析了PMGCT患者的DSS影响因素，从而构建了一个相对准确的列线图模型，但仍存在以下不足之处。首先，由于SEER数据库自身局限性，仅对具有放化疗信息的患者进行了登记，而对未做放化疗或者放化疗信息未知的患者登记为No/Unknown，另外因美国癌症联合委员会（American Joint Committee on Cancer, AJCC）肿瘤原发灶-淋巴结-转移（tumor-node-metastasis, TNM）分期非PMGCT主流分期评价标准^[8]^，且SEER数据库AJCC TNM分期数据缺失较大，故未将放化疗信息和AJCC TNM分期纳入到本次研究中，从而导致本研究样本数据有限。目前有一篇基于SEER数据库构建PMGCT患者OS列线图的文献^[25]^，与之相比，本文对数据进行了精简未将登记模糊的放化疗信息纳入DSS预后分析。其次，SEER数据库未纳入PMGCT患者的基础疾病状况、炎症指标、甲胎蛋白（alpha-fetoprotein, AFP）、β-人绒毛膜促性腺激素（β-human chorionic gonadotrophin, β-HCG）等指标，故本研究未对其进行分析。再次，由于PMGCT的发病率较低，病例数量较少，所以无法提供内部、外部验证。最后，SEER数据库所包含的病例主要来自美国，缺少中国等亚洲人的资料，缺乏一定普遍性。如果能够实现对上述内容的进一步验证，预后模型将更精准，更适用于我国人群。

综上所述，PMGCT是一种最常见的性腺外生殖细胞瘤，非精原细胞瘤组织学类型、未进行手术治疗、年龄>40岁、肿瘤大小≥15 cm是影响PMGCT患者DSS的独立危险因素。基于上述独立危险因素构建的列线图可以准确、直观、个体化地对PMGCT患者DSS进行预测，为临床医生评估PMGCT预后提供参考依据。
